# The anti-cancer effect and mechanism of animal scale-derived extract on malignant melanoma cells

**DOI:** 10.1038/s41598-023-39742-x

**Published:** 2023-08-02

**Authors:** Lanni Song, Chen Li, Jia Yu, Yixin Yang, Xuechen Tian, Siew Woh Choo

**Affiliations:** 1 Wenzhou Municipal Key Lab for Applied Biomedical and Biopharmaceutical Informatics, College of Science and Technology, Wenzhou-Kean University, Wenzhou, 325060 Zhejiang Province China; 2 Zhejiang Bioinformatics International Science and Technology Cooperation Center, Wenzhou-Kean University, Wenzhou, 325060 Zhejiang Province China; 3 Department of Biology, Wenzhou-Kean University, 88 Daxue Road, Ouhai, Wenzhou, 325060 Zhejiang Province China

**Keywords:** Cancer, Computational biology and bioinformatics, Drug discovery

## Abstract

Melanoma is a type of cancer with abnormal proliferation of melanocytes and is one of the most diagnosed cancer types. In traditional Chinese medicine, pangolin scales have been used to treat various diseases, including human cancers. However, its efficacy has not been scientifically proven. Here we studied the anticancer effect and mechanism of pangolin scale extract (PSE) on melanoma cell lines using scientific approaches. Our cell viability assay shows that PSE exhibits up to approximately 50–80% inhibition on SK-MEL-103 and A375 melanoma cell lines. Mechanically, PSE inhibits melanoma cell proliferation, migration, and causes changes in cell morphology. The apoptosis assay showed a significant chromosomal condensation inside the PSE-treated melanoma cells. The sequencing and analysis of A375 melanoma cell transcriptomes revealed 3077 differentially expressed genes in the 6 h treatment group and 8027 differentially expressed genes in the 72 h treatment group. Transcriptome analysis suggests that PSE may cause cell cycle arrest in melanoma cells and promote apoptosis mainly by up-regulating the p53 signaling pathway and down-regulating the PI3K-Akt signaling pathway. In this study, the anticancer effect of PSE was demonstrated by molecular biological means. PSE shows a significant inhibition effect on melanoma cell proliferation and cell migration in vitro, causes cell cycle arrest and promotes apoptosis through p53 and PI3K-AKT pathways. This study provides better insights into the anti-cancer efficacy and underlying mechanism of PSE and a theoretical basis for mining anticancer compounds or the development of new treatments for melanoma in the future. It is worth noting that this study does not advocate the use of the pangolin scale for disease treatment, but only to confirm its usefulness from a scientific research perspective and to encourage subsequent research around the development of active compounds to replace pangolin scales to achieve the conservation of this endangered species.

## Introduction

In the United States, there are over 5000 cases of cancer diagnosed each day, and cancer is still the second leading cause of human death in 2022, approximately 1700 death will be caused by cancer. The most extraordinarily diagnosed cancer type in the US could be lung cancer, colorectum cancer, breast cancer in women, prostate cancer in men, and melanoma^1^. Tumors are thought to be an abnormal proliferation of cells caused by genetic mutations, which are metastatic and can invade surrounding tissues or spread throughout the body through blood circulation^2,3^. Studies have found that some cancers may be inherited (e.g., melanoma, pancreatic cancer)^4,5^ and cancers may be induced by the environment and poor lifestyle habits, such as ultraviolet light (e.g., melanoma)^6^, viruses (e.g., cervical cancer)^7^, smoking (e.g., lung cancer)^8^, diet and drink (e.g., prostate cancer)^9^. The main treatment options for cancer are inseparable from surgery, chemotherapy, radiation therapy, and immunotherapy, they might effectively kill cancer cells, but the lack of targeting of cancer cells and drug resistance issues also make them do great harm to normal tissues and cells. Thus, the ultimate goal of cancer treatment research is to be able to specifically kill cancer cells without harming normal cells and tissues, whether through targeted therapy drugs or gene therapy of immune cells^10^.

Melanoma is a type of cancer with abnormal proliferation of melanocytes, where the skin produces the UV-absorbing pigment, melanin. It was estimated that nearly 100,000 people will be diagnosed with melanoma and with about 7650 cases of death in the US, and over 8000 new cases in China with a significantly higher mortality rate^1,11^. This may be due to the fact that melanoma receives less attention and awareness of its prevention in China, resulting in failure to intervene in the early stages of its development. The decreased mortality rate of malignant melanoma in the United States is mainly due to the strengthening of social prevention of melanoma, and the mortality rate of patients diagnosed with advanced melanoma is still high^12,13^.

The treatment of melanoma mainly relies on surgical resection and chemotherapy^14,15^. Dacarbazine is the mainly used chemotherapy agent recently, however, the response rate of dacarbazine analyzed in research shows not over 30%^16,17^. Besides, the side effects of dacarbazine could be severe with nausea and vomiting^18^. BRAF mutations are present in 80% of melanoma mutations, so targeting the BRAF gene is an important strategy for melanoma treatment. Vemurafenib and dabrafenib are two BRAF inhibitors but are still facing the drug resistance problem. Since immunotherapy entered the world, Interleukin 2 (IL-2) was the first immunotherapy drug approved by FDA, but it was highly toxic and only 6% of patients were cured^19^. Immune checkpoint inhibitors were introduced in 2011 and provided a good treatment strategy for the treatment of melanoma^20^. For example, ipilimumab, the anti-CTLA-4 antibody; Nivolumab and pembrolizumab, the anti-PD-1 antibodies are three important immune checkpoint inhibitors^21^. However, they can still induce serious side effects of inflammation conditions, and patients face high treatment costs^22^. Therefore, it is still important to discover new melanoma treatments, providing more effective and less recurrence-resistant treatment with fewer side effects is the main research direction.

Traditional Chinese Medicine (TCM) has been used in China for thousands of years and has a wide range of uses^23^. There is many proven effective Chinese medicine in the direction of anti-cancer, and anti-inflammatory^24,25^. TCM usually utilizes naturally occurring medicinal ingredients in the form of extracts, and thus their doses are usually not very large and have a relatively long course of treatment, but a smaller side effect. With molecular biology and “omics” becoming the mainstream biological research approach, the analysis of the potency and composition of TCM and their mechanisms have been gradually improved^26^. For instance, Zhai, Zhang^27^ introduced an anti-cancer compound, β-Elemene, extracted from Curcuma Rhizoma. Yan, Liu^28^ verified the molecular mechanism and main signaling pathway of Huaier aqueous extract against cervical cancer in vitro. In addition, some TCM ingredients have been found to affect gene expression or target specific genes. For example, curcumin modulates the p53 signaling pathway to exert its anti-cancer properties and it can also induce cancer cell apoptosis via regulating the Bax/Bcl-2 expression level^29,30^. TCM has also been proven to regulate the immune system positively, thus TCM is more frequently used as adjuvant therapy in combination with immunotherapy^31^. The pangolin scale is a TCM that has a high medicinal value and has been used to treat various diseases including cancer, inflammation-related diseases, leukopenia, and hyperlipidemia for hundreds of years ago^32^. However, the efficacy of pangolin scales in cancer treatment has not been scientifically proven, and its mechanism remains unclear.

Here we used scientific methods to study the anticancer effects and molecular mechanisms of pangolin scale extract (PSE) using A375 and SK-MEL-103 human malignant melanoma cell lines as models and exploring the anti-cancer mechanism of PSE at the transcriptome level through performing RNA-Seq to A375 melanoma cell line.

## Results

### PSE inhibits melanoma cell proliferation

**Figure 1:**
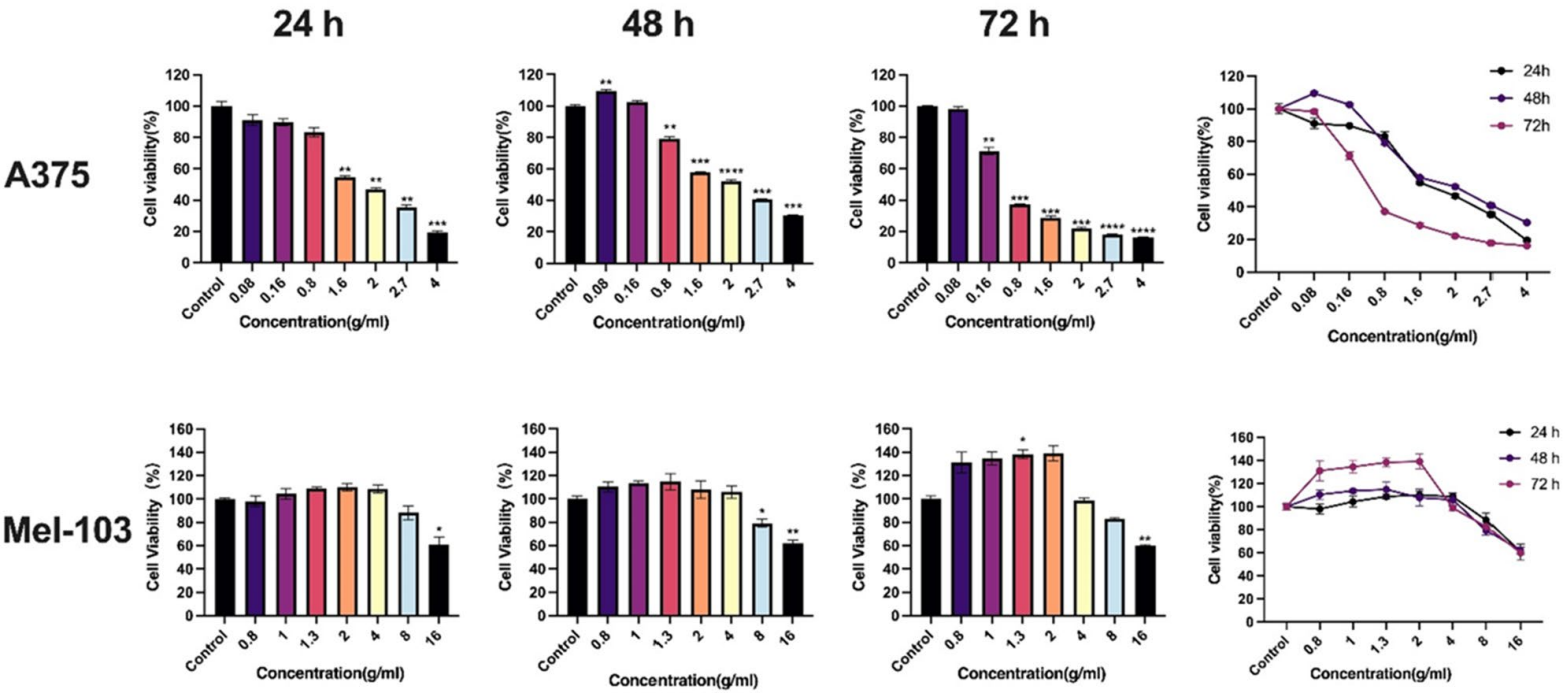
Cell viability assay. Effects of PSE on the proliferation of A375 and SK-MEL-103 melanoma cell lines were evaluated by CCK-8 assay. The viability of A375 cells was measured after treatment with gradient-diluted PSE for 24 h, 48 h, 72 h. The melanoma cell viability inhibitory effect of PSE showed a time- and concentration-dependent manner. Mean ± SEM shows the bar in graph. (*P < 0.05, **P < 0.01, ***P < 0.0005, ****P < 0.0001).

To determine melanoma cell viability under the treatment of PSE, a CCK-8 cell viability assay was performed. 8 g/ml and 16 g/ml PSE were first used to verify its tumor suppressive effect on melanoma cells. After preliminary testing, we found that PSE at this concentration has a strong melanoma suppressive efficiency. In order to find the lowest concentration that would ensure inhibition efficiency, we performed gradient dilutions of PSE to optimize the PSE concentration. Melanoma cells were treated with PSE that had undergone a gradient dilution of 0.08–16 g/ml for 24, 48, and 72 h, respectively. Our data showed a significant inhibitory effect of PSE on the viability of melanoma cells in a time- and concentration-dependent manner (Fig. 1). For instance, A375 and SK-MEL-103 cells showed an optimal inhibition effect under 72 h PSE treatment. The cell viability of A375 melanoma cells was approximately 20% (the rate of inhibition = 80%) after treatment with 4 g/ml of PSE for 72 h. SK-MEL-103 achieved about 60% cell viability, respectively.

### PSE induces apoptosis and reduces cell numbers of melanoma

**Figure 2:**
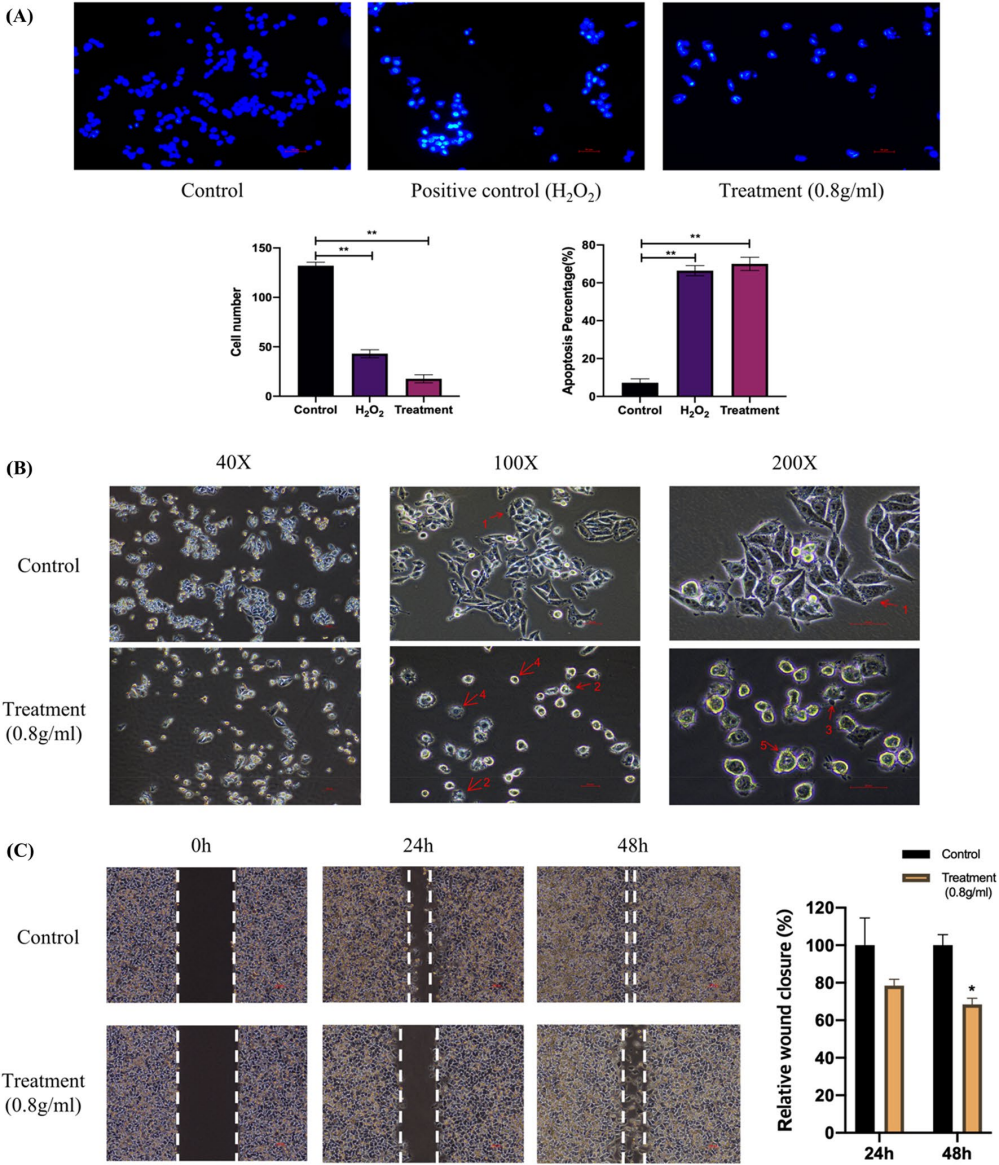
Anti-cancer effect of PSE on A375 melanoma cell line in vitro. (**A**) PSE induces apoptosis in A375 melanoma cells. A375 cells were treated with the complete medium; 5% H2O2; 0.8 g/ml PSE. Fluorescent staining of chromosomes was observed in the treatment group indicating the apoptotic process. The apoptotic rate and cell numbers of each group were shown in a bar chart. (**B**) PSE causes a morphological change in melanoma cells. 1: normal cells; 2: membrane blebbing; 3: apoptotic body; 4: cell shrinkage; 5: nuclear expansion. (**C**) PSE inhibits cell migration and invasion. The inhibition of cell migration was measured with the following formula: Relative wound closure = (wound area at 0 h–wound area at 24 h)/(wound area at 0 h). (*P < 0.05, **P < 0.01, ***P < 0.0005, ****P < 0.00005).

To examine whether PSE inhibits the growth of melanoma cells or kills them, the Hoechst 33,258 fluorescence staining assay was performed and compared the apoptotic activity of the treatment group (treated melanoma cells with 0.8 g/ml PSE for 72 h) with the control group (without treatment). The PSE-treated group showed a decreased cell number compared to the control group (e.g., 18 cells in treatment vs 132 cells in control) (Fig. 2A). The apoptotic rate of the treatment group reached 70% of cells, which was significantly higher than controls (Fig. 2A). In addition, it can be observed in the PSE-treated group that the nuclear part that was fluorescently stained appeared to be incomplete, and some of the fluorescence was in the fragmented form (Fig. 2A), suggesting that nuclear fragmentation might have occurred in the cells after treatment.

### PSE causes morphological changes in melanoma cells

To investigate whether PSE can alter the morphology of melanoma cells and thus affect cell function, the morphology of PSE-treated melanoma cells with that of cells in the normal stage by an inverted microscope was compared. In the control group, melanoma cells were shuttle-shaped with clear borders, the nuclei were observed, and the cells were tightly packed together. In contrast, the cell morphology was shrunk into a round shape, small in size, with large nuclei and blurred borders, and membrane blebbing and apoptotic body were observed at the periphery in the treatment group (Fig. 2B).

### PSE can inhibit melanoma cell migration

To further investigate whether PSE affects the migration of melanoma cells, a cell wound healing assay was performed to verify the cell migration inhibitory effect of scale extract. After 24 h treatment with 0.8 g/ml PSE, the treatment group showed a tendency to inhibit cell migration (Fig. 2C). After 48 h treatment, the treatment group showed a wider wound area with a migration rate of 68% compared to controls (Fig. 2C).

### RNA extraction and integrity

To give better insights into the underlying mechanisms or pathways in melanoma cells response to PSE, RNA-Seq experiments were performed to study the changes in gene expression profiles. Total RNA was extracted from control melanoma cells (without treatment) and melanoma cells treated with 0.8 g/ml of PSE for 6 h (TA group) and 72 h (TB group), respectively. All RNA samples met the sequencing requirements of the Illumina sequencing platform, with RNA integrity numbers (RIN) all greater than 8.0 (Supplementary Table 2).

### Transcriptome sequencing, read quality control, and normalization

After RNA extraction and library construction, all libraries were sequenced on the Illumina sequencing technology platform, and raw reads were obtained. During the sequencing process, a small number of reads were sequenced with splice sequences or the quality of 3’ ends was too low (Supplementary Table 3). Therefore, the raw data were preprocessed and the low-quality reads were filtered by the Cutadapt software^33^ to remove contamination and junction sequences. After preprocessing, approximately 1.062 billion clean reads of about 148 bp read length were obtained. The Q30 percentage of the filtered clean data reached approximately 95%, indicating that the quality of sequencing data is high and suitable for downstream analyses (Supplementary Table 4).

After clean reads were generated, reads were mapped on the human reference genome sequence (GRCh38.p13 version; Accession number: GCA_000001405.28). Our data showed that at least 91% of the clean reads were mapped onto the reference genome for each sample, of which about 85% were uniquely mapped reads (Supplementary Table 5). Our results indicate the high quality of our data and are suitable for subsequent analyses.

### Principle component analysis (PCA)

**Figure 3:**
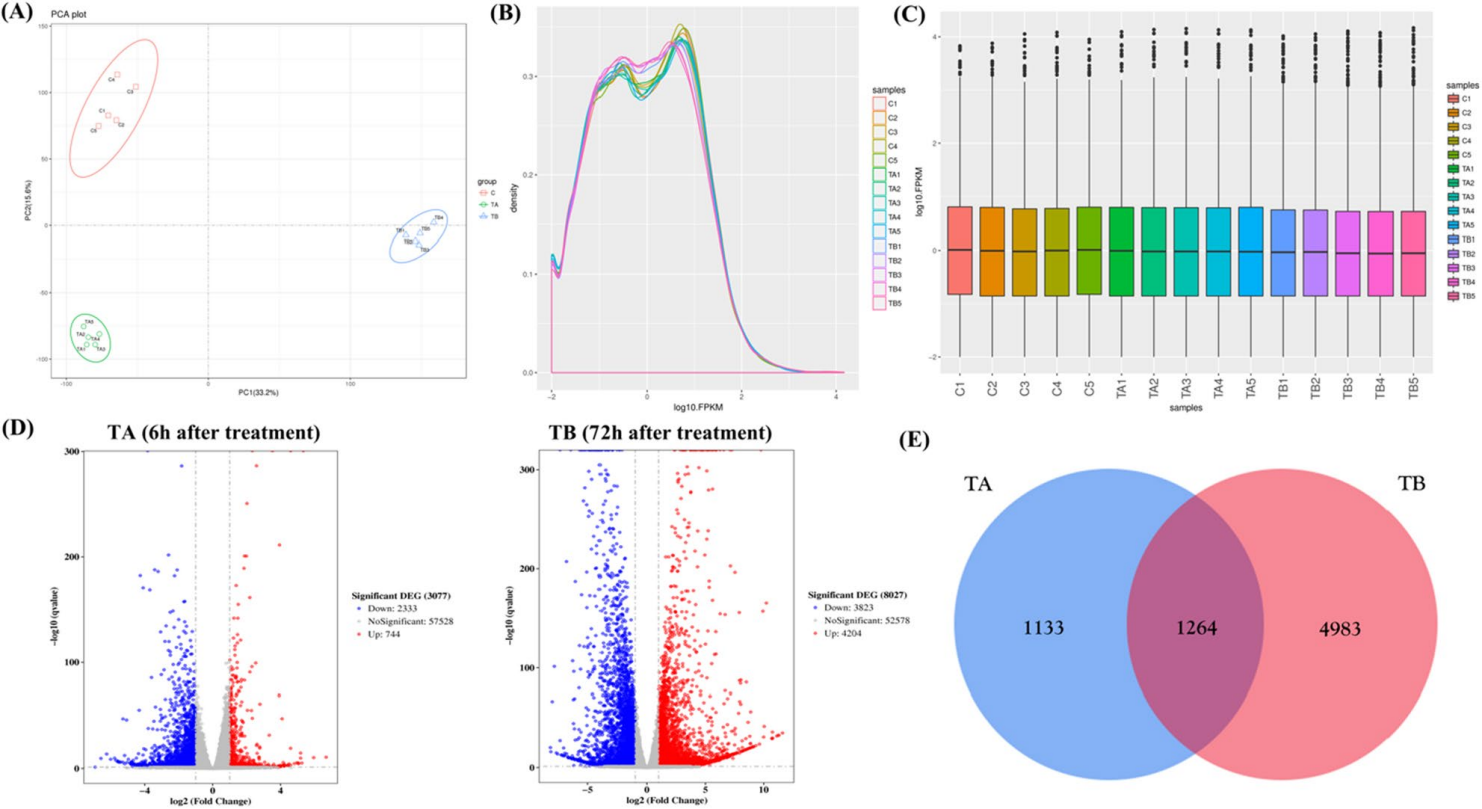
PCA analysis and differential gene expression analysis. (**A**) The distribution of the groups on the plot is significantly different, indicating the differences between different treatment groups. (**B**) FPKM distribution map, the abscissa is log10(FPKM), and the ordinate is the density of genes. (**C**) FPKM box chart, abscissa is the sample name, vertical coordinate is log10(FPKM), and the box plot of each region pairs five statistics (from top to down are maximum, upper quartile, median, lower quartile, and minimum value). (**D**) Among all the DEGs, red dots are significantly up-regulated genes, while blue dots are significantly down-regulated genes. The abscissa represents the fold change in gene expression and the ordinate represents the statistical significance of the difference in gene expression variation. (**E**) Venn plot of differential genes, Venn plots showed the number of genes that are unique to two different time points (TA and TB).

To verify the relationship and size of variation between the three groups of samples, this study performed a PCA on 15 samples (Fig. 3A). The position of each point represents the value of the sample on each principal component. The result indicates that there were significant intergroup differences between the three groups of samples, the intra-group differences were small, and the samples were clustered. This data showed that there could be a significant difference between the expression profiles of the three datasets.

### Differential expression gene analysis

To perform differential gene analysis, the gene expression levels between the three sample groups were normalized (Fig. 3B–C). After normalization, all samples have similar expression distribution or the mean value was at a similar level, suggesting that the normalization was successful and suitable for comparison. To identify differentially expressed genes (DEGs), the expression profiles of TA and TB groups were compared with the control group, respectively. In the C versus TA group, DEG comparison results, we identified 3077 DEGs, including 744 up-regulated genes and 2333 down-regulated genes (Fig. 3D); In the C vs TB group, we identified 8027 DEGs, including 4204 up-regulated genes and 3823 down-regulated genes (Fig. 3D). By comparing the two sets of DEGs, there were 1557 DEGs from C versus TA that coincided with the genes from C-TB (Fig. 3E). However, there was a large number of TA-specific (1133 genes) or TB-specific genes (4983 genes), suggesting that although there is some commonality, the gene expression profiles at 6 h and 72 h after treatment were quite different. A possible explanation is that different sets of genes or pathways might be regulated at the two different time points after PSE treatment.

### Functional enrichment analysis

**Figure 4:**
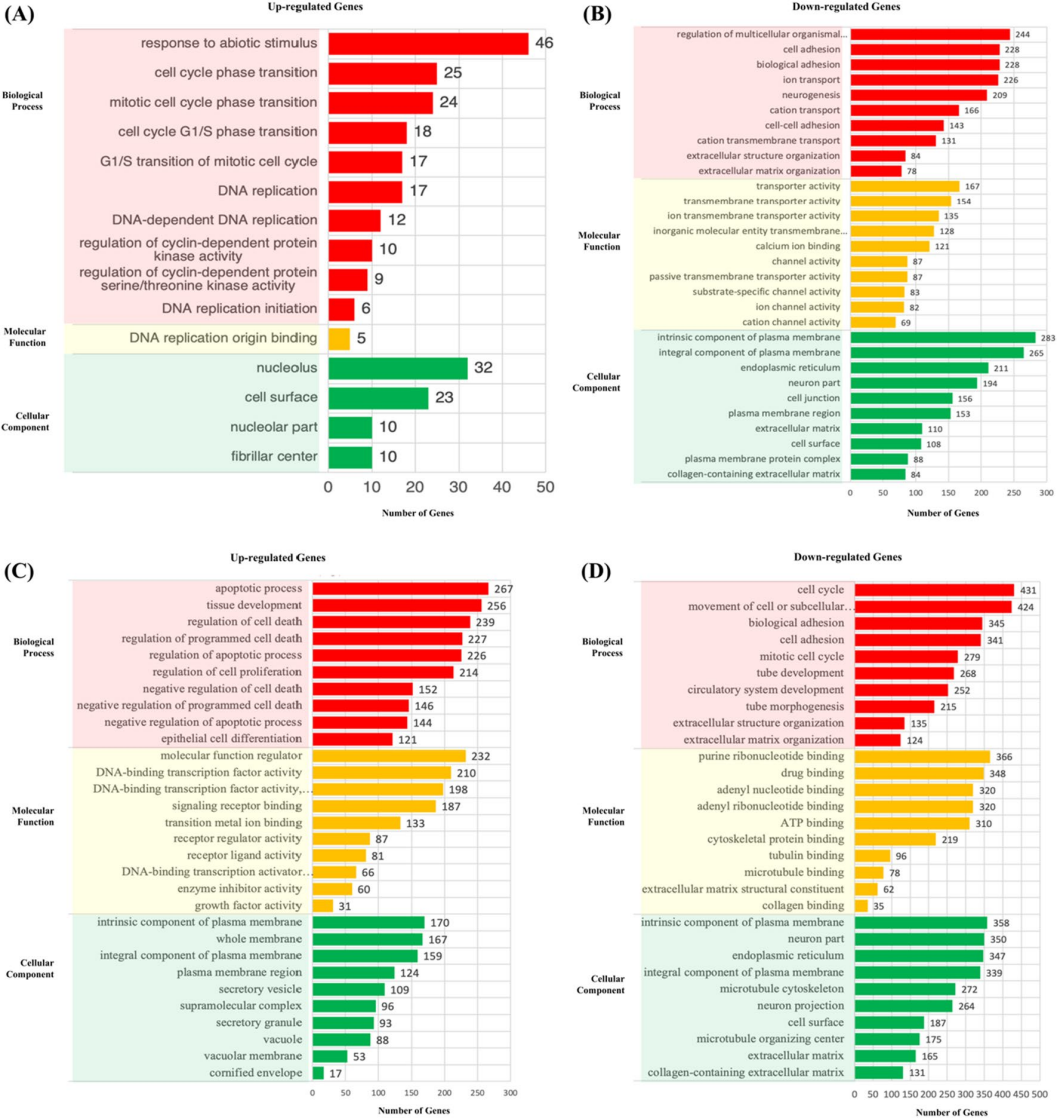
Gene Ontology enrichment analysis. The top 10 GO terms of biological process, molecular function, and cellular component are selected according to FDR sorting and summarized. The bars were ranked according to the number of genes enriched to the corresponding GO terms. (**A**,**B**) up- and down-regulated GO terms enriched from DEGs of C-TA comparison. (**C**,**D**) up- and down-regulated GO terms enriched from DEGs of C-TB comparison.

To have better insights into the functions of DEGs, Gene ontology (GO) enrichment analysis of these DEGs was performed using WebGestalt^34^. The up-and-down-regulated genes of TA and TB groups were analyzed separately. Our results showed that the top 10 enriched biological processes ranked based on FDR from up-regulated DEGs of TA are in response to abiotic stimulus, cell cycle G1/S phase transition, G1/S transition of mitotic cell cycle, DNA replication, cell cycle phase transition, mitotic cell cycle phase transition, regulation of cyclin-dependent protein kinase activity, DNA-dependent DNA replication, DNA replication initiation and regulation of cyclin-dependent protein serine/threonine kinase activity (Fig. 4A). The top 10 enriched biological processes from down-regulated DEGs of TA are regulation of multicellular organismal development, ion transport neurogenesis, biological adhesion, cell adhesion, cation transport, cation transmembrane transport, cell–cell adhesion, extracellular structure organization and extracellular matrix organization (Fig. 4B). The up-regulated gene-enriched biological process in TA is associated with cell cycle and mitosis, while the down-regulated gene-enriched biological process is associated with extracellular structure and cell adhesion. In the GO analysis of DEGs from TB group, a total of 369 up-regulated biological process, and 1131 down-regulated biological process was enriched. Among them, the top 10 enriched up-regulated terms are related to biological processes such as regulation of the apoptotic process, regulation of programmed cell death, regulation of cell death, apoptotic process, negative regulation of programmed cell death, negative regulated of the apoptotic process, tissue development, negative regulation of cell death, epithelial cell differentiation, regulation of cell proliferation (Fig. 4C). The top 10 enriched down-regulated terms are movement of cell or subcellular component, cell cycle, biological adhesion, cell adhesion, circulatory system development, tube development, mitotic cell cycle, tube morphogenesis, extracellular structure organization, and extracellular matrix organization (Fig. 4D). The biological processes enriched in the TB group related to up-regulation of cell apoptosis and the down-regulation of the cell cycle.

**Figure 5:**
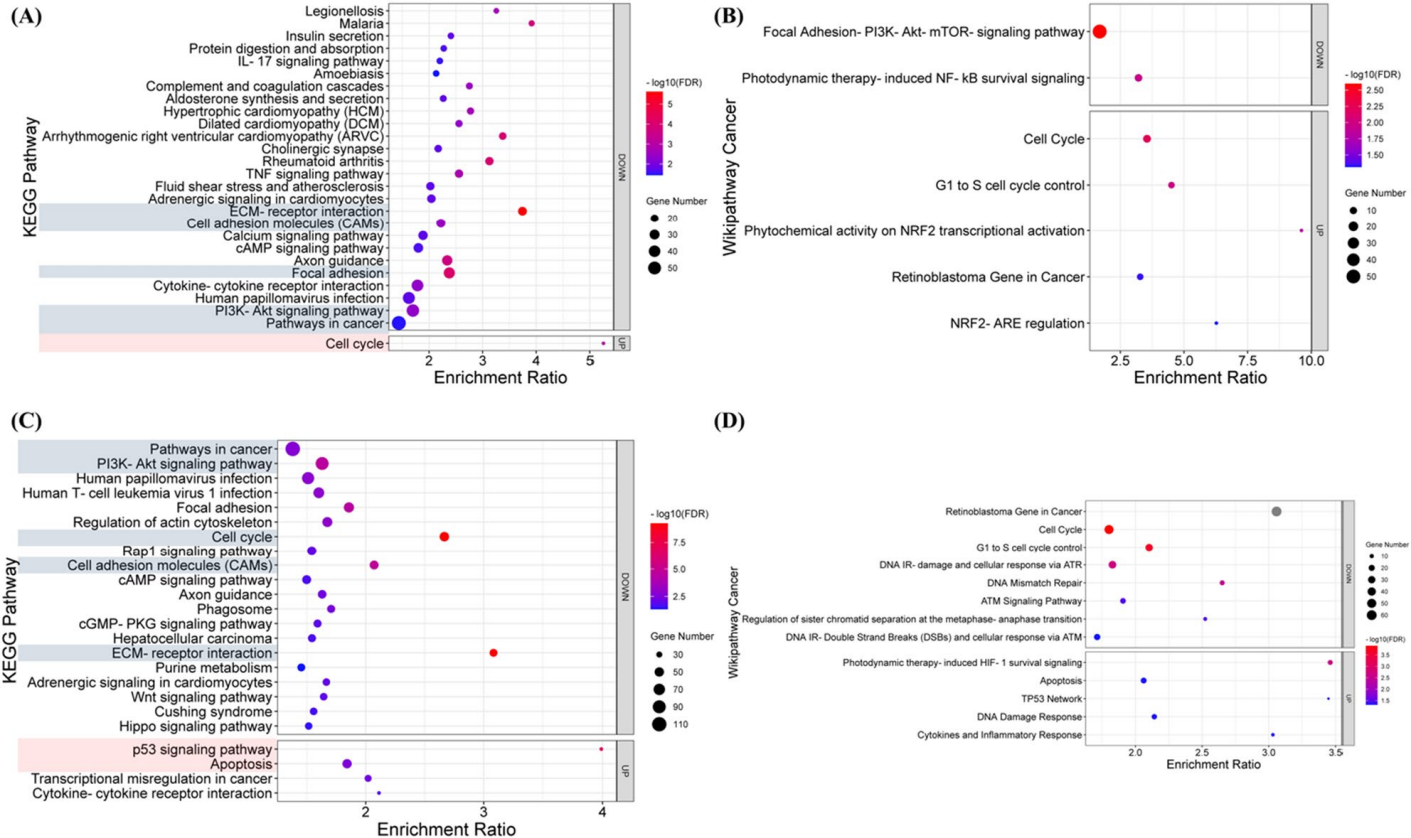
KEGG and Wikipathway cancer pathway enrichment analyses. (**A**,**B**) pathway enrichment of TA; (**C**,**D**) pathway enrichment of TB. The up- and down-regulated DEGs were analyzed respectively, the horizontal axis of bubble plot indicates the enrichment ratio of each pathway, the size of each bubble indicates the number of genes enriched to each pathway, and the color of the bubble reflects the value of -log10 (FDR).

To understand the main modalities and signaling pathways of melanoma cells affected by PSE, KEGG, and cancer-associated Wikipathway enrichment analyses were performed, respectively. Our KEGG pathway enrichment analysis showed one up-regulated signaling pathway (i.e., cell cycle pathway) and 26 down-regulated signaling pathways (i.e., ECM-receptor interaction) at 6 h after PSE treatment (Fig. 5A). Other down-regulated pathways include focal adhesion, TNF signaling pathway, cytokine-cytokine receptor interaction, PI3K-Akt signaling pathway, calcium signaling pathway, cAMP signaling pathway, IL-17 signaling pathway, pathways in cancer; and some pathways associated with other diseases, such as rheumatoid arthritis, malaria, legionellosis, etc. The Wikipathway Cancer result of TA showed five up-regulated pathways, phytochemical activity on NRF2 transcriptional activation, NRF2-ARE regulation, G1 to S cell cycle control, cell cycle and retinoblastoma gene in cancer; and two down-regulated pathways, that are, photodynamic therapy-induced NF-kB survival signaling pathway and focal adhesion-PI3K-Akt-mTOR-signaling pathway (Fig. 5B). In the KEGG results of TB, four pathways were up-regulated, they were p53 signaling pathway, cytokine-cytokine receptor interaction, transcriptional misregulation in cancer, and apoptosis. And 64 pathways were down-regulated including cell cycle, DNA replication, ECM-receptor interaction, PI3K-Akt signaling pathway, etc. Notably, the cytokine-cytokine receptor interaction pathway was down-regulated in TA but up-regulated in TB (Fig. 5C). In the cancer-associated wikipathway result, there were five up-regulated pathways: photodynamic therapy-induced HIF-1 survival signaling, apoptosis, TP53 network, DNA damage response, and cytokines and inflammatory response; meanwhile, eight pathways were down-regulated including retinoblastoma gene in cancer, cell cycle, G1 to S cell cycle control, DNA IR-damage and cellular response via ATR, DNA mismatch repair, ATM signaling pathway, regulation of sister chromatid separation at the metaphase-anaphase transition, DNA IR-DBSs and cellular response via ATM (Fig. 5D).

### Validation of DEGs by RT-qPCR

Through transcriptome analysis, several signaling pathways that may be affected by PSE have been identified, such as apoptosis, PI3K-AKT, p53 signaling pathway, cell cycle, etc. To verify the expression of some major differentially expressed genes in melanoma cells, and to verify the accuracy of transcriptome analysis as well, we selected nine genes for RT-qPCR verification. Among o them are p21, BAX, BCL-2, PI3k, Akt, CDK6, CCNB2, CCL3 and SPINK1. In order to have higher sensitivity, we selected the TB group (0.8 g/ml PSE, 72 h) showing higher fold change values for RT-qPCR verification and selected GAPDH as the internal reference. Although CCL3 and SPINK1 are not part of the signaling pathway of interest, they had high fold change in RNA-Seq result, so they were included to validate the accuracy of RNA-Seq.

**Figure 6:**
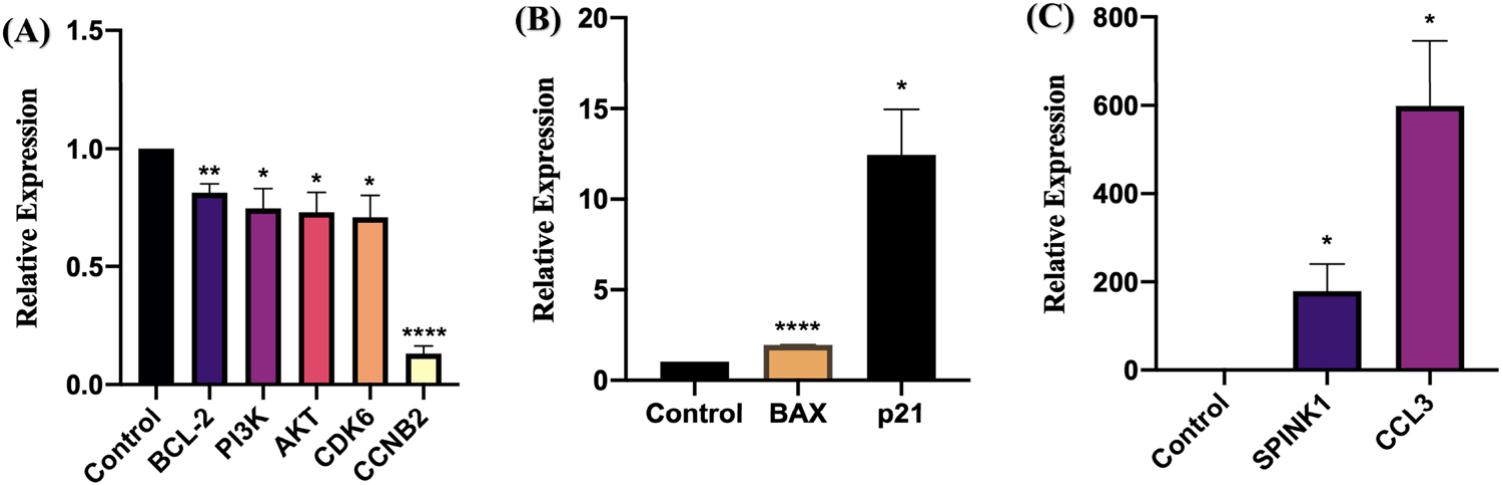
Gene expression assay under 72 h of PSE treatment by RT-qPCR. (**A**) Genes with significant under-expression. (**B**,**C**) Genes with significant over-expression. GAPDH housekeeping gene was used as an internal reference. Mean ± SEM shows the bar in graph. (*P < 0.05, **P < 0.01, ***P < 0.0005, ****P < 0.0001).

Our data showed that the nine validated genes possessed the same expression trend in RT-qPCR results as in RNA-Seq data (Fig. 6). That is, BAX, p21, CCL3, and SPINK1 were over-expressed, while the remaining genes were under-expressed. Therefore, the results from RT-qPCR are consistent with the RNA-Seq data.

## Discussion and conclusion

Here we first-time explore the anti-cancer effect and mechanism of PSE using two human malignant melanoma cell lines as models. Our data showed that the anti-cancer effect of PSE was positively correlated with the duration and concentration of action, and reduced the viability of melanoma cells. Besides, PSE-treated melanoma cells exhibited morphological changes such as membrane blebbing, apoptotic body, cell shrinkage, and nuclear expansion. Altered cell morphology may be associated with cell adhesion and cell migration^35^. Our wound-healing assay suggests that PSE inhibited the migration rate of melanoma cells. Mechanically, PSE kills melanoma cells by inducing apoptosis.

Our RNA-Seq analysis showed an enrichment of upregulated DEGs in biological processes related to the cell cycle, especially G1/S phase transitions and DNA replication at 6 h after the treatment with PSE, indicating that melanoma cells are likely still growing at 6 h after the treatment. However, on the cell membrane, some changes caused by PSE were still observed. At this early stage, PSE down-regulated the expression of genes involved in biological processes related to cell adhesion and cell-extracellular structure. It is possible that the changes in cell morphology might be related to cell migration or caused by apoptosis (cell death). Besides, TA showed significant down-regulation of genes in the focal adhesion-PI3K-Akt-mTOR-signaling pathway. Among this pathway, DEGs were distributed in the upstream part of the pathway, such as ECM-receptor interaction-related genes and growth factors. We cannot rule out the possibility that the growth of melanoma cells might have begun to be slowly inhibited, although the cell line is still in a state of continuous proliferation. The result of functional enrichment of TB is more intuitive. Our data showed the up-regulation of the apoptosis process and down-regulation of the cell cycle process, likely through the up-regulation of the p53 signaling pathway and the down-regulation of the PI3K-Akt signaling pathway. Their results suggest that PSE might have completely induced apoptosis and suppressed the cell cycle in melanoma cells at 72 h after the treatment. It should be noted that the key gene in the p53 signaling pathway, TP53, is a tumor suppressor gene commonly found in tumors, so it is often up-regulated and it is an important strategy in many cancer treatments to induce cell apoptosis^36^. However, our transcriptomic data showed that p53 was under-expressed, meanwhile, in the downstream part of this pathway, a large number of p53-regulated genes, such as p21, BAX, bcl-2, are up-regulated, likely promoting the initiation of apoptosis. Therefore, it can be suspected that there were other strategies to induce the overall up-regulation of the p53 signaling pathway, as well as inducing apoptosis of melanoma. The other interesting signaling pathway, the PI3K-Akt signaling pathway, has a role in regulating cell proliferation, therefore, its down-regulation might be the main pathway for PSE to inhibit melanoma proliferation. In this pathway, two key genes, PI3K and Akt, were significantly under-expressed, and their downstream pathways are also in line with expectations.

Melanoma is a cancer type with the most mutations and targeting different genes is an important strategy for the treatment of melanoma^37^. Among them, since the BRAF gene mutation of the MEK pathway accounts for 60% of melanoma mutations, the existing drugs and most of the targeted therapy in the trial are designed to act as BRAF inhibitors, such as vemurafenib and dabrafenib^38^. The PI3K-Akt pathway is another important target for melanoma treatment to solve the resistance of BRAF inhibitors or MEK inhibitors^39^. Some drugs or studies targeting the PI3K-Akt pathway are mainly classified into PI3K inhibitors, Akt inhibitors, PI3K isoform inhibitors, and mTORC1 inhibitors^40^. Pedini, De Luca^41^ demonstrated that a PI3k-Akt signaling pathway inhibitor, PIK-75, combined with vemurafenib shows a further anti-cancer effect on drug-resistant melanoma in vitro and in vivo. Van Dort, Galban^42^ synthesized a dual-effect inhibitor based on PI3K inhibitors and MEK inhibitors and inhibited the in vitro viability of cancer cells. Our functional gene enrichment analysis demonstrated a down-regulation of the PI3K-Akt signaling pathway and down-regulated PI3K gene and Akt gene. This may be the role of a single component in a PSE or it may be a combination of ingredients, but it is a good illustration of the anti-cancer potential and mechanism of PSE, therefore it deserves more studies in the future.

Furthermore, TP53 is a well-known tumor suppressor gene that encodes the p53 protein which is a transcription factor response under stress conditions including oncologic overexpression, hypoxia, DNA damage, and senescence^43^. Under stress conditions, p53 is activated and continues to activate downstream pathways to influence the biological processes within the cells including cell cycle, apoptosis, cell differentiation, and senescence^44^. There are many p53-based treatment strategies in existing cancer treatments, including high expression of the p53 gene with mdm2 inhibitors or MDMX inhibitors. However, p53 has been found to mutate in many cancer cells, restore the wild-type p53 or reduce the stability of mutant p53 and activate the downstream of p53 signaling pathway are some of the strategies of cancer therapy. Foster, Coffey^45^ first demonstrated a small-molecule CP-31398, that will up-regulate the p21 (CDKN1A) gene, which is an important downstream gene in the p53 signaling pathway. In our study, the transcriptomic profile showed an up-regulation of the p53 signaling pathway. However, it should note that the TP53 gene itself was down-regulated, which may be because of the overexpression of its negative regulator MDM2 gene. This suggests that there may be other ways to replace the role of p53 and induce downstream activation of the p53 signaling pathway, including positive regulation of genes downstream of the p53 pathway (i.e., p21, BAX, Bcl-2, and CDK6). P21 is a cyclin-dependent kinase inhibitor and is usually activated by p53^46^. P21 will bind to cyclin-dependent kinase 2 (CDK2), CDK1 and inhibits their ability, causing a G1 cell cycle arrest^47^. BAX and Bcl-2 are a set of genes that regulate cell mitochondrial apoptosis. BAX is a pro-apoptotic protein, while Bcl-2 is an anti-apoptotic protein^48^.

Studying the changes in the transcriptome of melanoma cells at 6 h may help to understand how genes respond to PSE in the early stage of the treatment and also probably identify direct targets (possible therapeutic targets) regulated by the PSE. However, there are still a large number of DEGs (i.e., about 3000 genes) at 6 h after the treatment, making the identification of direct target genes more complicated. It would be interesting to identify DEGs at an earlier time point (i.e., 2–4 h) to identify more direct targets regulated by PSE in the future. The large number of DEGs at 6 h after the treatment indicates that PSE has caused a series of responses in a short period of time. One of the possible explanations is that PSE contains many components that may affect many biological processes or pathways.

In this study, although we have shown that PSE exhibits anti-cancer activity, we do not know which active compound contributes to this effect. These compounds may individually exert anticancer effects or produce synergistic effects. It is also an advantage of TCM. The synergistic effect produced by the combination of different components can often reduce the toxicity and side effects caused by the medicinal use of a single component. However, it is still important to discover the most effective combination of ingredients. Therefore, future research will aim to identify, synthesize, and screen for anti-cancer compounds (e.g., peptides and metabolites) in PSE. In the meanwhile, we hope to find potential targets for active ingredients in future work, and further verify the mechanism of the anti-cancer effect of PSE on melanoma through functional analysis. It is worth noting that the pangolin is an endangered species that should be valued and protected. We do not advocate any direct usage of pangolin scales in disease treatment. From a scientific research perspective, we encourage subsequent research around the development of active ingredients to replace pangolin scales in diseases to achieve the conservation of this endangered species.

In conclusion, this study successfully studied the anticancer activity of PSE and the underlying mechanism against melanoma cell lines. Our data showed that PSE could inhibit the growth of two melanoma cell lines, supported by evidence from cell viability. Mechanically, PSE inhibits the proliferation and migration of melanoma cells and induces apoptosis. Whole-transcriptome analysis reveals that PSE may cause cell cycle arrest in melanoma cells and promote apoptosis mainly by upregulating the p53 signaling pathway and down-regulating the PI3K-Akt signaling pathway. The study provides a fundamental basis for mining anticancer compounds, and new research directions, and may improve the current management of melanoma in the future.

## Materials and methods

### PSE preparation

Pangolin scales used in this study were legally purchased from a pharmaceutical company (Tongrentang, China). This study was an in vitro experiment using traditional medicine products in commercial cell lines and did not involve any animal ethics-related living animal. Pangolin scales were ground into powder using a grinder, and 8 g and 16 g pangolin scale powder were weighed into separate conical flasks with ddH_2_O. The flask was placed on a magnetic stirring heating table and the pangolin scale was extracted at 60 °C for 24 h, and the speed of the magnetic stirring bar was adjusted according to the volume of liquid to prevent the powder from settling. The extract was then filtered using a 2 mm filter and the liquid phase was separated. The extract was then dispensed into 2 ml centrifuge tubes and concentrated at − 80 °C using a freezing vacuum centrifuge (Labconco Freezone, USA) until the liquid evaporated completely. Then 1 ml of sterilized water was used to dissolve the precipitate in the centrifuge tubes and was filtered again to obtain the final extract. The concentration of the PSE solution at this stage was determined as 8 g/ml and 16 g/ml, respectively. The PSE was stored at − 20 °C for future usage. The PSE used in subsequent experiments was gradually diluted from the original concentration.

### Cell culture

A375, SK-MEL-103 malignant melanoma cell lines were cultured with Dulbecco Modified Eagle Medium (Gibco, USA) containing 10% of Fetal Bovine Serum (Gibco, USA) and 1% of penicillin–streptomycin (Gibco, USA). Cells were placed in a 37 °C incubator with a 5% of CO_2_ atmosphere.

### Cell viability assay

To verify the in vitro inhibitory effect of PSE on melanoma and to further optimize the concentration and duration of action of the PSE for optimal anti-cancer effects, a cell viability assay was performed using a CCK-8 cell counting kit (Dojindo, Japan). Cells were harvested when they reached 80–90% of density. Cells were seeded into a 96-well culture plate (NEST Biotechnology Co.LTD., China) with a concentration of 2000 cells/well. Cells were cultured overnight for adhesion. After the cells fully adhered, the original medium was removed and 90ul of fresh complete medium was added, followed by 10ul of gradient-diluted PSE. 96-well plate with melanoma cells was placed into a 37 °C incubator for 24, 48, and 72 h respectively. 10 ul of CCK-8 was added into each well and incubated for 2 h and the optical density at 450 nm was measured using a microplate reader (Varioskan flash, Thermo Scientific, USA). The experiment was replicated three times.

### Cell migration assay

To verify the inhibitory effect of PSE on A375 melanoma cell migration in vitro, a wound-healing assay was performed to calculate the cell migration rate under treatment. A 2-well culture insert (Ibidi, Germany) was placed in the middle of a 20 mm culture plate, 5 × 10^4^ cells within 70ul of complete medium were applied to each well, and the outer area was filled with 1.8 ml of complete medium. Cultured at 37 °C and 5% CO_2_ for 24 h results in a confluent layer. After the attachment, the culture insert was gently removed using sterile tweezers and the cell wound was washed twice using PBS. 2 ml of complete medium containing 200 ul of H_2_O (for the control group) or optimized concentration of PSE (for the treatment group) were added to the culture dish. Photographs of the cell wounds were taken at 0, 12, 24, 36, and 48 h of incubation with a phase contrast inverted microscope (100× magnification). The cell wound area was counted by image j software. The cell migration rate was calculated as follows: Wound closure % = [1 (wound area at T_t_/wound area at T_0_) × 100%], where Tt is the time after wounding and T0 is the time immediately after wounding. The experiment was replicated three times.

### Cell apoptosis assay

Cell apoptosis was examined with Hoechst 33,258 staining kit (Beyotime, China). A 10 cm coverslips were placed into a 6-well plate, and 4 × 10^4^ of A375 cells were seeded into each well. Cells were cultured overnight, then the medium was removed followed by the cells being washed twice in PBS. Cells were then treated with 0.8 g/ml of PSE for the treatment group and the same volume of H_2_O for the control group for 72 h. Before fixation, 200 ul of 5% of H_2_O_2_ was added to the positive control group for 20 min to induce cell apoptosis. After stimulating the cell apoptosis, 0.5 ml of the fixative solution was added for 20 min. Cells then were washed and stained according to the manufacturer's instructions. The coverslips were placed on glass slides dripped with an anti-quenching solution for observation. Cells were then observed and photographed by an inverted fluorescence microscope (Nikon, MA100N, Japan). To reduce subjectivity as much as possible, we took random pictures of the several spots of each dish with a fluorescence microscope. Subsequently, we randomly selected three pictures per dish (a total of 9 photos per group) and used ImageJ software to count the number of cells and the number of cells producing fluorescence, respectively^49,50^. The experiment was replicated three times.

### Examination of cell morphology

To investigate whether PSE will cause a morphological change in the A375 melanoma cell line, 6 × 10^4^ cells were seeded into a 60 mm cell culture plate and were cultured overnight. After cell attachment, cells were washed by PBS twice, and the medium was refreshed with 2.7 ml of fresh complete medium and 300 ul of H_2_O for the control group and 300 ul of PSE with optimized concentration for the treatment group and both of them were cultured for the optimized duration. The morphological change of melanoma cells was detected with a phase-contrast inverted microscope (Nikon, Japan) at 100X and 200X. The experiment was replicated three times^51,52^.

### RNA extraction and integrity analysis

To analyze the mechanism of action of PSE on A375 melanoma cell line at different time points, and study the initiation of the cellular response against PSE, the cells were divided into three groups: control group, 6 h PSE-treated group, 72 h PSE-treated group. The total RNA was extracted and their integrity was assessed based on the following protocols. 1 × 10^5^ of A375 melanoma cells were seeded into a 60 mm cell culture dish with 5 ml of complete medium. After being cultured overnight, cells in 6 h and 72 h treatment groups were treated with 0.8 g/ml of PSE for 6 h and 72 h respectively, and the same volume of H_2_O was added into the control group, also cultured for 72 h. The medium was collected into a 15 ml centrifuge tube, and cells attached to the dish were scraped off with a spatula with 1 ml of PBS and collected into the centrifuge tube. Cells were centrifuged under 1000 rpm for 3 min to pellet. 1 ml of Trizol (Ambion, USA) was added and cells were transferred into a 1.5 ml EP tube and stood for 5 min at room temperature to fully lyse. 200ul of Chloroform was added, shaken vigorously, and stood for 10 min at room temperature followed by a centrifuge at 13,000 rpm for 10 min. The aqueous phase was transferred to a fresh tube and 0.5 ml of isopropanol was added. Stood for 10 min at room temperature and centrifuged at 13,000 rpm for 10 min. The pellet was washed with 75% ethanol followed by centrifuging. After vortex and centrifuging, the RNA pellet was resuspended in DEPC-treated water. The concentration and purity of total RNA were inspected by Nanodrop 2000 (Thermo Fisher Scientific Inc., USA) and the RNA integrity was evaluated by the Agilent 2200 Bioanalyzer (Agilent Technologies, USA).

### Library construction and whole-transcriptome sequencing

The library preparation was performed using 1ug of total RNA. The isolation of poly(A) mRNA was performed utilizing Oligo (dT) beads. Divalent cations and a high temperature were used to fragment the mRNA. Random Primers were used for priming. The first and second strands of cDNA were produced. After that, the purified double-strand cDNA was treated in one reaction to repair both ends and add a dA-tail, followed by a T-A ligation to add adaptors to both ends. Afterward, DNA clean beads were used to size-select Adaptor-ligated DNA. Each sample was then amplified by PCR with P5 and P7 primers and products were evaluated. Then, according to the manufacturer’s instruction, libraries with various indexes were multiplexed and placed onto an Illumina Novaseq 6000 instrument for sequencing using a 2 × 150 paired-end sequencing strategy.

### Read preprocessing and mapping

Bcl2fastq (v2. 17.1.14, RRID: SCR_015058) (bcl2fastq Conversion Software, 2022) was utilized to perform image base calling and preliminary quality analysis on the original image data of the sequencing results, the original sequencing data were obtained, and the result was saved in the FASTQ file format. Cutadapt (v1.9.1, phred cutoff: 20, error rate:0.1, adapter overlap: 1 bp, min. length: 75, proportion of N: 0.1) was used to remove technical sequences, including adapters, PCR primers, or fragment thereof, and quality of bases lower than 20 (Phrep score).

### Principal component analysis

To visualize sample-to-sample distances, a principal component analysis was performed between three groups of data. The samples were projected onto the 2D plane and spread out in the two directions that account for the majority of the variations. The x-axis is the direction in which the samples were separated most and the value was written PC1. The y-axis is the direction in which samples were separated the second most and the value was written PC2. The percent of the total variance that is contained in the direction was printed on the axis label.

### Gene expression quantification, normalization, and differential expression analysis

Human reference genome (GRCh38.p13) sequences were downloaded from ENSEMBL. The reference genome sequence was indexed using Hisat2 (v2.0.1), and the clean data were aligned to the reference genome. Transcripts in FASTA format were first converted from a known gff annotation file and appropriately indexed. HTSeq (v0.6.1) assessed gene and isoform expression levels from the pair-end clean data using the file as a reference gene file. DESeq2 Bioconductor package, then, was used for analyzing differential expression genes between the control group and two treatment groups respectively. Among them, genes with fold change ≥ 2, Padj < 0.05 were considered differentially expressed genes (DEGs). For a more accurate follow-up analysis of DEGs, they were classified into up-regulated genes and down-regulated genes according to the positive and negative fold changes.

### GO annotation and KEGG enrichment analysis

All DEGs were submitted to the Gene Ontology Resource (http://www.geneontology.org) for Gene Ontology (GO) enrichment analysis. DEGs were assigned into gene ontology terms, which were characterized by three categories: biological process, molecular function, and cellular component. In addition, up- and down-regulated DEGs were mapped onto the KEGG (Kyoto Encyclopedia of Genes and Genomes) (https://www.genome.jp/kegg/) pathway and WikiPathway (https://www.wikipathways.org/index.php/WikiPathways) Cancer for pathway enrichment analysis. Both GO terms and KEGG pathway terms were selected if False Discovery Rate (FDR) < 0.05.

### RT-qPCR

To verify our transcriptome analysis results, several genes were selected for verification by real-time quantitative polymerase chain reaction (RT-qPCR), in order to test whether the displayed gene levels were consistent with the transcriptome data of the TB group. Primers were designed using NCBI Primer-BLAST (https://www.ncbi.nlm.nih.gov/tools/primer-blast/) and synthesized by Shanghai Sangon Biological Company (Supplementary Table 1). Cells were treated with 0.8 g/ml of PSE for 72 h, and total RNA was extracted using the same protocol introduced above and was reverse transcribed to cDNA according to the instructions of PrimerScriptTM RT reagent Kit (Takara, Japan). Add 4ul 5 × mix, 1ug RNA to the PCR tube, and finally make up to 20ul with RNase Free ddH2O. The reaction program is 37 °C for 15 min, 85 °C for 5 s, 4 °C; after the reaction, store at 4 °C. The qPCR was performed utilizing Hieff UNICON^®^ Universal Blue qPCR SYBR Green Master Mix (Yeasen, Shanghai) according to the manufacturer’s instructions. The RT-qPCR program was completed by QuantStudio™ 3 (Applied Biosystems, USA).

### Ethics approval and consent to participate

The material (scales) used in this study was purchased from a pharmaceutical company, and all experiments were carried out in vitro without involving any living animal. Therefore, this study may not apply to an ethics approval.

## Supplementary Information

Supplementary Tables.

## Data Availability

The RNA sequencing data can be accessed at the CNGB website (https://db.cngb.org/cnsa/) with an accession number CNP0003531, and the BioProject PRJNA885306 for National Center for Biotechnology Information (nih.gov). Other information in this study has been referred to in the supplementary tables.
